# Supplementary material for: Thermodynamic behavior of correlated electron-hole fluids in van der Waals heterostructures
Source: Nat Commun. 2023 Dec 13;14:8264. doi: 10.1038/s41467-023-43799-7 (PMC10719388; doi:10.1038/s41467-023-43799-7)
Supplement: Supplementary file 1 — Supplementary Information [file 41467_2023_43799_MOESM1_ESM.pdf]

Supplementary Information for

Thermodynamic behavior of correlated electron-hole fluids in van der Waals heterostructures

Ruishi Qi[1,2,†], Andrew Y. Joe[1,2,†,*], Zuocheng Zhang[1], Yongxin Zeng[3], Tiancheng Zheng[1,4], Qixin Feng[1,2], Jingxu Xie[2,5], Emma Regan[1,2,5], Zheyu Lu[2,5], Takashi Taniguchi[6], Kenji Watanabe[7], Sefaattin Tongay[8], Michael F. Crommie[1,2], Allan H. MacDonald[3], and Feng Wang[1,2,9,*]

[1]Department of Physics, University of California, Berkeley, CA 94720, USA.
[2]Materials Sciences Division, Lawrence Berkeley National Laboratory, Berkeley, CA 94720, USA.
[3]Department of Physics, University of Texas at Austin, Austin, Texas 78712, USA.
[4]School of Physical Sciences, University of Chinese Academy of Sciences, Beijing, China.
[5]Graduate Group in Applied Science and Technology, University of California at Berkeley, Berkeley, CA 94720, USA.
[6]International Center for Materials Nanoarchitectonics, National Institute for Materials Science, 1-1 Namiki, Tsukuba 305-0044, Japan.
[7]Research Center for Functional Materials, National Institute for Materials Science, 1-1 Namiki, Tsukuba 305-0044, Japan.
[8]School for Engineering of Matter, Transport and Energy, Arizona State University, Tempe, AZ 85287, USA.
[9]Kavli Energy NanoSciences Institute, University of California Berkeley and Lawrence Berkeley National Laboratory, Berkeley, CA 94720, USA.
[†]These authors contributed equally.
[*]To whom correspondence should be addressed: andrew.joe@ucr.edu, fengwang76@berkeley.edu

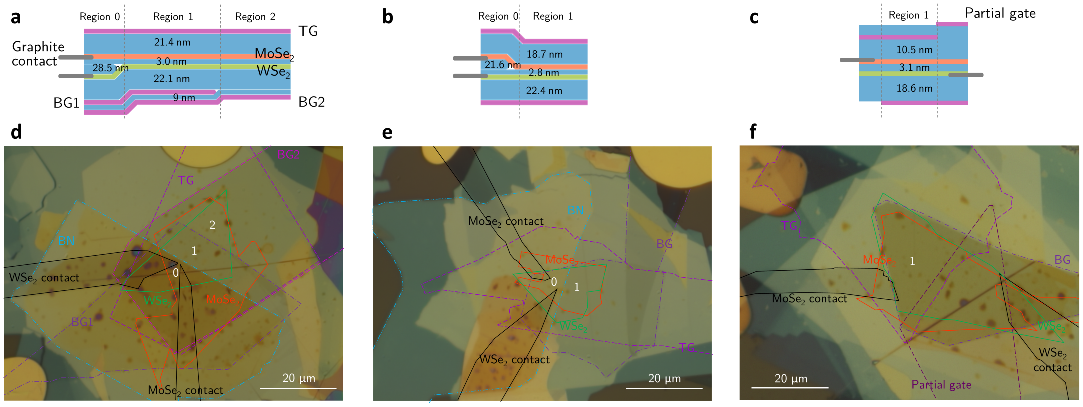

Supplementary Figure 1: Device structure and optical images. a-c Schematic cross section of devices D1 (a), D2 (b) and D3 (c). **d-f** Optical microscope images of the devices, with flake boundaries outlined.

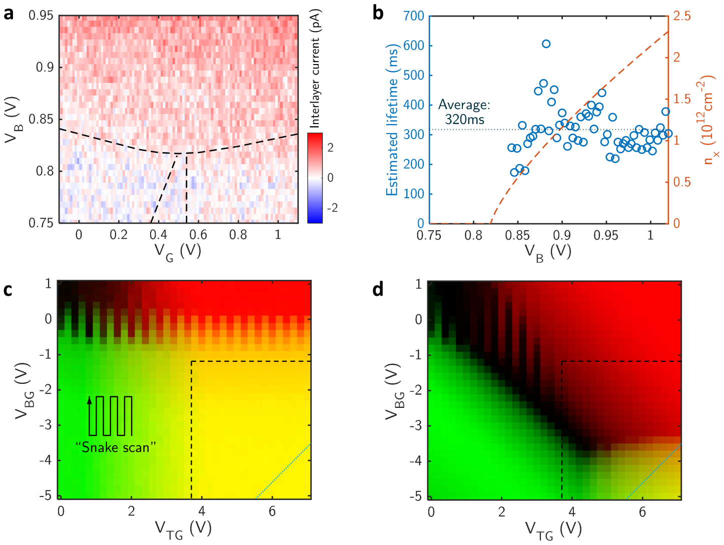

Supplementary Figure 2: Equilibrium electron-hole fluids. a Interlayer leakage current as a function of the bias and gate voltage. **b** Left axis: Estimated recombination lifetime calculated from the interlayer leakage current along $n_\mathrm{e} \approx n_\mathrm{h}$ line. Right axis: Interlayer exciton density along the same line cut. **c, d** Region 0 and region 1 doping phase diagram at constant $V_\mathrm{B} = 1$ V. Red and green channels of the image indicate electron and hole doping, respectively. The data is taken with alternating scan direction for neighboring columns (snake scan). Inside the dashed boxes, region 0 is at high doping for both layers, and region 1 shows no hysteresis. Blue dotted line indicates where the data in the main text is taken.

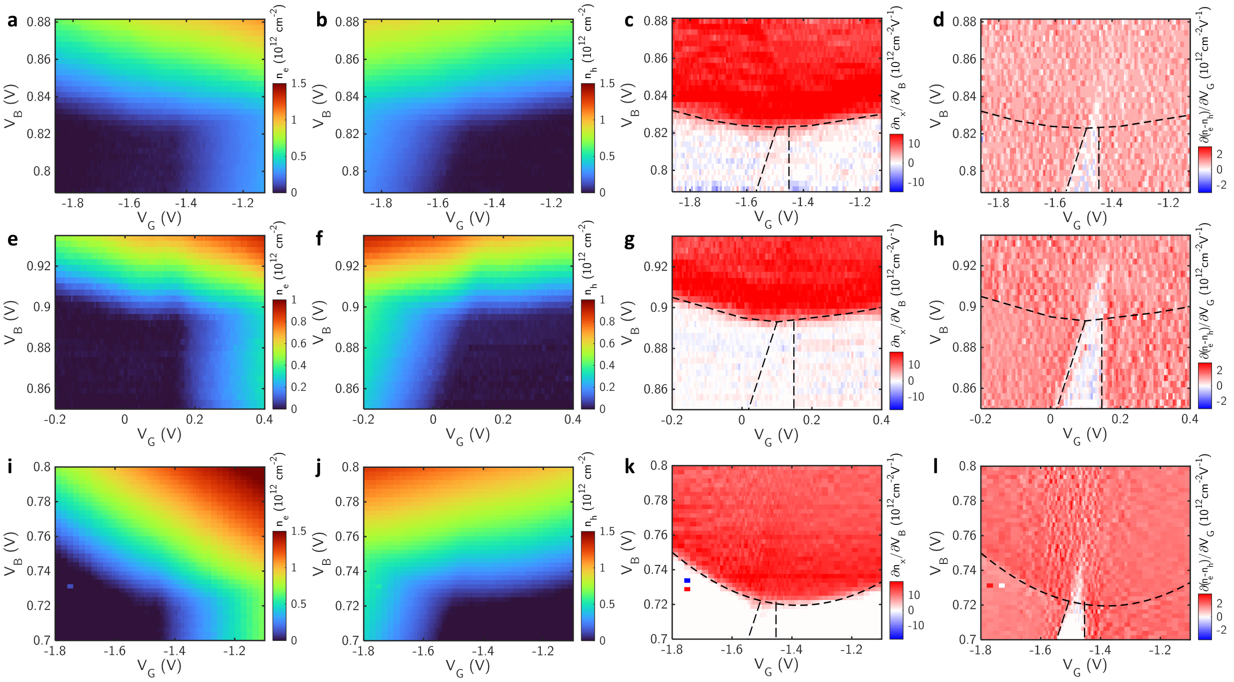

Supplementary Figure 3: Density maps from additional devices. a-d Measured electron density (a), hole density (b), exciton compressibility (c), and charge compressibility (d) from D1 region 2 with $V_\Delta = 13$ V. **e-h** Same data from D2 with $V_\Delta = 9$ V. **i-l** Same data from D3 with $V_\Delta = 8$ V.

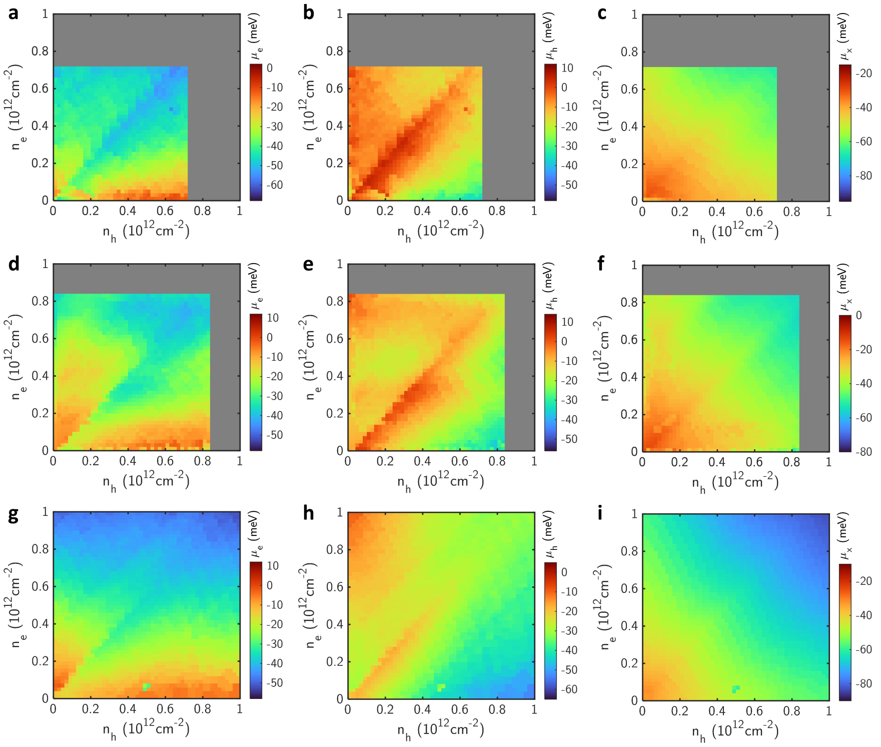

Supplementary Figure 4: Experimental chemical potential from additional devices. a-c Electron (a), hole (b), and exciton (c) chemical potentials extracted from D1 region 2. **d-f** Same data from D2. **g-i** Same data from D3.

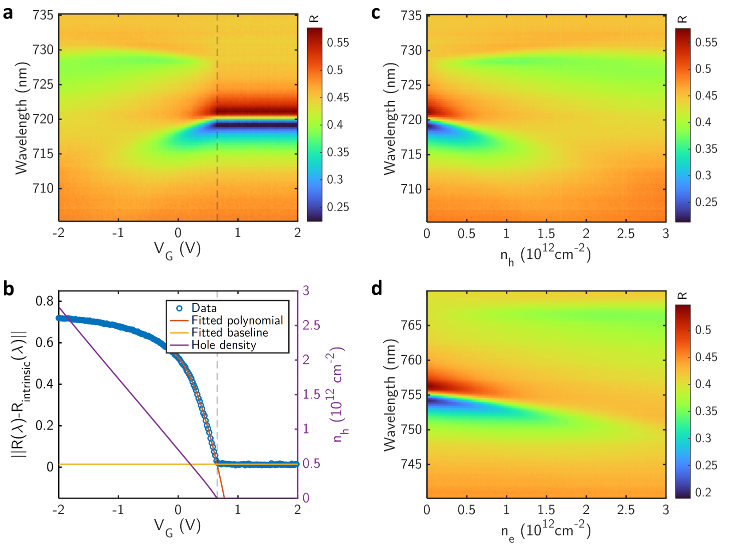

Supplementary Figure 5: Carrier density calibration. **a** Gate dependence of the absorption spectrum at $V_B = 1$ V, $V_\Delta = 7$ V. The type-II band gap is not closed under this condition, so at most one layer is doped. **b** Determination of the critical voltage that the chemical potential is on the valence band edge. Blue scatters, change of reflection spectrum relative to charge neutrality. Yellow and red curves are fitted baseline and fitted polynomial curves that capture the initial change of the spectrum after the start of the doping. The vertical dashed line is the determined voltage at which the doping begins. Purple line is the calibrated hole density based on the model described in Methods section. **c, d** WSe_2 and $MoSe_2$ reflection spectra as a function of the charge density.

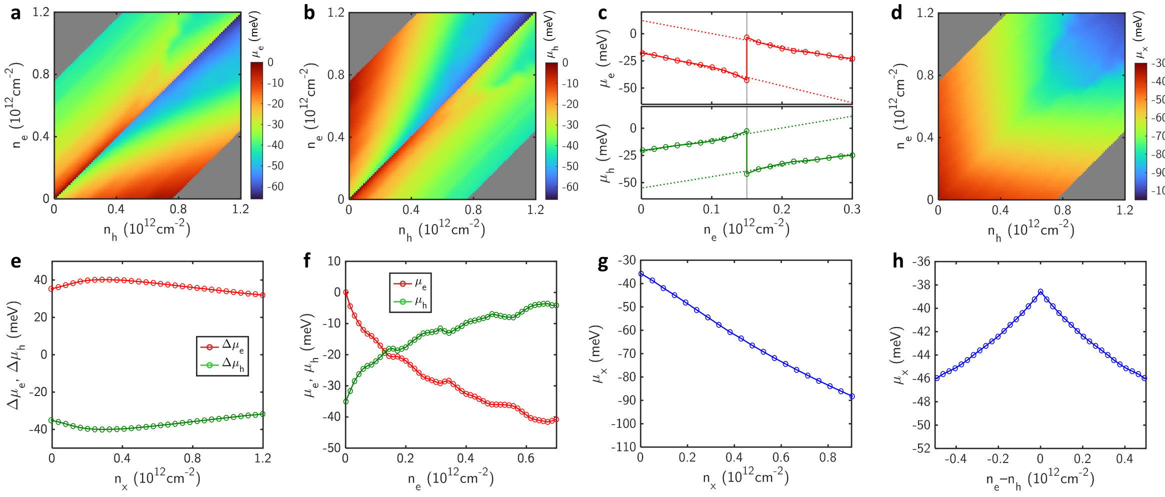

Supplementary Figure 6: Mean-field theory calculation. **a-b** Calculated chemical potential maps for electrons (a) and holes (b). **c** Line cut of the electron and hole chemical potentials at fixed hole density $n_h = 0.15 \times 10^{12}\text{cm}^{-2}$. **d** Calculated exciton chemical potential map $\mu_x = \mu_e + \mu_h$. **e** Chemical potential jump at net charge neutrality for different exciton densities. The mean-field theory does not capture the Mott transition due to lack of consideration of screening. **f** Electron chemical potential $\mu_e(n_e, n_h = 0)$ and hole chemical potential $\mu_h(n_e, n_h = 0)$ as a function of electron density, keeping the hole density zero. **g** Exciton chemical potential as a function of exciton density, keeping electron and hole densities equal. **h** Exciton chemical potential as a function of unpaired charge density $n_e - n_h$, keeping exciton density $n_x = 0.05 \times 10^{12}\text{cm}^{-2}$ constant.

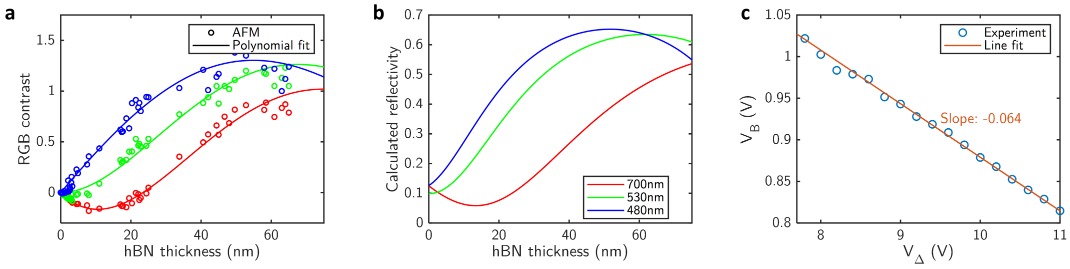

Supplementary Figure 7: hBN thickness calibration. **a** Scatter plot of the measured red, green, and blue (RGB) channels of the hBN optical contrast for different measured hBN thicknesses. The solid lines are 4th order polynomial fits to the experimental points. **b** Calculated reflectivity based on Fresnel equations. The system consists of (from top to bottom) semi-infinite air, hBN with variable thickness, 90 nm SiO_2 and semi-infinite Si. Normal incidence is assumed. **c** Bias voltage required to close the band gap as a function of V_Δ. The slope gives the negative ratio of thin hBN thickness to the total hBN thickness (see Methods for details).
